# Professional roles of general practitioners, community pharmacists and specialist providers in collaborative medication deprescribing - a qualitative study

**DOI:** 10.1186/s12875-020-01255-1

**Published:** 2020-09-04

**Authors:** Navina Gerlach, Matthias Michiels-Corsten, Annika Viniol, Tanja Schleef, Ulrike Junius-Walker, Olaf Krause, Norbert Donner-Banzhoff

**Affiliations:** 1 Department of General Practice, University of Marburg, Karl-von-Frisch-Straße 4, D-35043 Marburg, Germany; 2 Hannover Medical School, Institute of General Practice, Carl-Neuberg-Straße 1, D-30625 Hannover, Germany

**Keywords:** General practice, Polypharmacy, Deprescriptions, Intersectoral collaboration, Multimorbidity, Medication therapy management, Medication optimization, Qualitative research.

## Abstract

**Background:**

Collaborative care approaches between general practitioners (GPs) and pharmacists have received international recognition for medication optimization and deprescribing efforts. Although specialist providers have been shown to influence deprescribing, their profession so far remains omitted from collaborative care approaches for medication optimization. Similarly, while explorative studies on role perception and collaboration between GPs and pharmacists grow, interaction with specialists for medication optimization is neglected. Our qualitative study therefore aims to explore GPs’, community pharmacists’ and specialist providers’ role perceptions of deprescribing, and to identify interpersonal as well as structural factors that may influence collaborative medication optimization approaches.

**Method:**

Seven focus-group discussions with GPs, community pharmacists and community specialists were conducted in Hesse and Lower Saxony, Germany. The topic guide focused on views and experiences with deprescribing with special attention to inter-professional collaboration. We conducted conventional content analysis and conceptualized emerging themes using the Theoretical Domains Framework.

**Results:**

Twenty-six GPs, four community pharmacists and three community specialists took part in the study. The main themes corresponded to the four domains ‘Social/professional role and identity’ (1), ‘Social influences’ (2), ‘Reinforcement’ (3) and ´Environmental context and resources’ (4) which were further described by beliefs statements, that is inductively developed key messages. For (1), GPs emerged as central medication managers while pharmacists and specialists were assigned confined or subordinated tasks in deprescribing. Social influences (2) encompassed patients’ trust in GPs as a support, while specialists and pharmacists were believed to threaten GPs’ role and deprescribing attempts. Reinforcements (3) negatively affected GPs’ and pharmacists’ effort in medication optimization by social reprimand and lacking reward. Environmental context (4) impeded deprescribing efforts by deficient reimbursement and resources as well as fragmentation of care, while informational and gate-keeping resources remained underutilized.

**Conclusion:**

Understanding stakeholders’ role perceptions on collaborative deprescribing is a prerequisite for joint approaches to medication management. We found that clear definition and dissemination of roles and responsibilities are premise for avoiding intergroup conflicts. Role performance and collaboration must further be supported by structural factors like adequate reimbursement, resources and a transparent continuity of care.

## Background

As the prevalence of polypharmacy increases in old and multimorbid populations, deprescribing high-risk or unnecessary medication has received growing attention [1, 2]. Deprescribing, defined as the proactive, systematic process of identifying and discontinuing inappropriate medicines [3], however is challenging for various reasons. Firstly, therapeutic recommendations of single-disease guidelines often influence or even conflict each other, exacerbating medication management for multimorbid patients [4–7]. Furthermore, providers from different healthcare settings and levels-of-care interact as multimorbid patients frequently visit various health-professionals [8–10]. Consequences of this entail poor communication and unclear responsibilities between providers, as well as experiences of hierarchy and fragmentation of care. Accordingly, transparency and even quality of pharmaceutical treatment may be impaired [4, 11–15]. Even when medication discontinuation is indicated and agreed upon by physician(s) and patient, however, the deprescribing process itself demands skills as well as time resources [10, 16]. Limitations in knowledge and evidence about deprescribing additionally foster insecurities [15].

Often, general practitioners (GPs) are considered gatekeepers for coordination and medication treatment of multimorbid patients [4, 15]. With the current demographic transition, however, this has created surmounting demand in primary care across developed healthcare systems [12, 16]. In the UK, Canada and Australia, initiatives responding to this workload challenge have piloted collaborative care approaches for optimized and efficient medication management [14]. These approaches commonly entail cooperation between GPs, optionally practice nurses, and community or ´clinical` pharmacists. In Australia, accredited pharmacists engage in Home Medication Reviews as well as Residential Medication Management Reviews to promote safe medication use. In Canada, similar medication reviews are conducted at primary care settings [15]. Medication reviews may include reconciliation of prescription errors or redundancies, medication evaluation for patient-specific aspects, and performance of medication interaction assessment [17]. Elsewhere, pharmacists furthermore engage in provision of prescriber education, patient counselling on medication management or lifestyle advice. Hitherto, physicians participating in several of these approaches adopted a substantial degree of pharmacists’ recommendations for medication change or deprescribing [13, 17, 18]. Though impact on clinical outcomes is difficult to detect from existing studies [19], collaborative care approaches revealed positive effects for patient-related, procedural and health-economic outcomes [13, 20–23]. In a well-observed prospective cohort study in the UK, delegation to clinical pharmacists reduced GP-time spent on key prescribing activities by 51%, equating to 4.9 h of work per week being released [13]. In sum, policy makers around the globe are recognizing the potential of pharmacists to reduce GP workload and optimize care, as well as the need to further extend integration of professions into regular primary care provision [20, 24].

In Germany, work overload is persisting among primary-care practitioners. Still, numbers of practicing GPs are exceeded by those of community specialists (CSs), who independently work in outpatient secondary-care practices. GPs in Germany have limited gate-keeping functions and their interaction with CSs concentrates on referrals which, however, rarely are compulsory [25]. Hence, community-specialist services not only are highly accessible for patients, but also reflect great therapeutic autonomy such as for medication provision. Even community pharmacists’ (CPs’) services are easily accessible and free-of-charge. Yet, in Germany, only physicians may prescribe medications. Despite CPs’ interest in enhancing service provision beyond pharmaceutical sales distribution, like by medication management tasks [26], no larger-scale collaborative primary-care approach with GPs and CPs has been prompted so far. Even with attention emerging about pharmacists’ potential in medication optimization and primary care cooperation [27], providers in Germany continue working rather isolated. This silo-focused healthcare approach has long been criticized for triggering medication therapy problems due to information loss [28–30]. Attempts to bridge information gaps by introducing sector-wide electronic health records however have failed for reasons of data protection.

Indeed, even specialist providers’ impact on deprescribing has been affirmed in several studies [9, 31–34]. More recently, even a cross-level perspective on deprescribing has increased, which addresses interaction and influences of different healthcare settings [10, 15]. Although attempts to combine the views of GPs, pharmacists and specialist providers have been made in qualitative research, their views on deprescribing have been inquired rather confined to their own profession [35–37]. Hence, a distinct focus on interdisciplinary collaboration between these groups is missing. For the German setting, the role of specialist providers for joint medication optimization efforts remains unobserved.

Our study therefore aims to explore GPs’, pharmacists’ and specialist providers’ perceptions of professional roles in deprescribing with a focus on inter-professional collaboration for medication optimization.

## Methods

### Design and setting

The study was approved by the Hannover Medical School Ethics committee and the University Marburg Ethics committee (No. 2326–2014, 160/15). This work is part of a larger study aiming to support deprescribing of unnecessary polypharmacy in the primary-care setting. Together with a systematic review on deprescribing instruments [38], this focus-group study constituted the exploration stage to inform the design of an electronically-supported deprescribing intervention among German GPs (results not published yet). For this purpose, we chose qualitative methods to get in-depth accounts on our study topics. Using focus-group discussions (FGDs) with GPs, pharmacists and specialists allowed us to access not only personal views and perceptions on these topics, but also social dynamics between stakeholders within and across the three professional groups. To receive information generated within a protected environment, we assembled GPs-only for half of the groups (FGD no. 1,2,5,6). For the remaining groups, we opened up for controversies and tensions in inter-professional exchange by inviting GPs and pharmacists (FGD 4), GPs and specialists (FGD 7) and all three professions (FGD 3). The discussions oriented on a pre-defined topic guide developed by the research team in iterative meetings. The topic guide aimed to explore participants’ views of and experiences with deprescribing, motivations and barriers to deprescribe, as well as views and experiences of cooperation between professional groups in this matter (topic guide is available as supplementary material).

Participants were recruited via academic research networks at the study sites in Hesse and Lower Saxony, Germany. We used purposeful sampling to respect variations in practice site (urban vs. rural setting), years of work experience and sex. Recruitment was performed via written invitation letters entailing response forms, followed by phone-calls to interested responders. The response rate for participation of GPs, CPs and CSs respectively was 33%; 20 and 10%. All discussions were conducted at university facilities in 2017. After questions were answered and written consent was obtained, the FGDs were moderated by a tandem of facilitators including each a physician and a social scientist from the research team (MMC, NG, TS, OK). Three of the four facilitators (MMC, NG and TS) had experience in the conduct of FGD and all were experienced in qualitative research. The discussions were recorded in audio and video to allow for assignment of speaker to each quote. As no further novel themes emerged in discussions with GPs, and no more CPs and CSs could be recruited, data collection was concluded.

### Participants and sample descriptions

Seven FGDs with in total 33 participants were conducted. Of those, 26 participants were GPs, four CPs and three CSs. Although we attempted to gather specialists from different disciplines, only cardiologists and one neurologist agreed to participate, however the neurologist dropped out short-term due to illness. Of the GPs, 12 participants were female and 14 male, of the CPs, three were female and one male and all CSs were male. Age ranged from 38 to 65 years and work experience ranged from 10 to 34 years. The participants’ work environment displayed the intended heterogeneity with 18% working in a large city, 33% in a medium-sized town, 27% in small towns and 21% in rural settings. Of the GPs, five worked in single practices, 20 in group practices and one in a health center. Focus group sizes varied between three and seven participants, and the discussions lasted between 85 and 117 min (mean 104 min.) The FGDs were held in the regions of Hesse (groups no. 1–4) and Lower Saxony (groups no. 5–7) in Germany.

### Analysis and presentation of findings

All discussions were transcribed verbatim and coded using qualitative software program (MaxQDA version 12). We performed conventional content analysis as described by Hsieh and Shannon [39] to avoid imposing preconceived theories but instead allow for categories to inductively emerge from the data itself.

All transcripts were independently coded by two researches with medical and social-scientific background (NG and MMC) in an iterative coding process. Discrepancies in coding were resolved in discussions whilst their organization in categories was undertaken in close collaboration.

After development of categories, the central themes were organized using the Theoretical Domains Framework (TDF) for better conceptualization of core dimensions. The TDF has been developed by synthesis of 33 theories basing on 128 theoretical constructs to assist analysis of cognitive, affective, social and environmental influences on behavior [40]. The 14 identified domains cover individual factors as well as the physical and social environment. For the present data material, the four domains ‘Social/professional role and identity’, ‘Social influences’, ‘Reinforcement’ and ‘Environmental context and resources’ were judged most relevant to further clarify the developed categories and themes. While social/professional role and identity depicts a set of displayed personal qualities in a work setting including roles and boundaries, social influences entail component constructs such as social norms, social pressure, power and intergroup conflict, but also social support. For reinforcement, response promoting stimuli like rewards, incentives, punishment and sanctions are understood. Lastly, environmental context and resources denote any circumstances that reveal stressors, resources, barriers and facilitators [40].

In qualitative research, the formulation of specific ‘belief statements’ has been suggested to further apprehend data within the applicable domains of the TDF [41]. A belief statement is an underlying idea about a problem and/or influence on the target implementation problem that has been uttered in a collection of responses [42]. Hence, belief statements convey the quintessence of the guiding themes in each conceptual domain.

To abstract the relations of domains to each other in a deprescribing context, the four domains were organized into a continuum ranging from individual to structural factors. For rendering detailed insight in the qualitative material while maintaining readability, we summarize quotations in (Tables 1, 2, 3 and 4). These comprehensive collections allow for validity assessment and offer information on consistency and divergence of records. Generally, a quote’s speaker is indicated by abbreviation of his or her professional group, participant ID and sex, e.g. ‘GP1M’ for General practitioner 1, male.

**Table 1:** The domain of professional role and identity

Professional role and identity as related to deprescribing		
Belief (as expressed by professional group)	Quote No.	Quote and speaker (indicating professional group, participant ID and sex e.g. GP1M = General practitioner 1, male)
GPs are the central medication managers (GP, CS, CP)	1	Facilitator 1: How is it in general, who is responsible for medication? The GPs, or the specialists? GP25F: We are. The GPs.
	2	CS33M: (...) all that usually is transferred to the central manager of the patient, the GP, who should check the medication.
	3	CS32M: In my understanding, the GP basically has the management supremacy. There is no other way. In a time when specialist groups become smaller and smaller and more and more specialized, where always more single medications emerge, these ´blinkered specialists` are no longer able to know what’s really necessary.
	4	CP21M: That’s why you, the GP, act as an interface – not the specialist. Because the latter only sees his own specialty. The ophthalmologist considers eye drops for glaucoma. But he doesn’t take note of what else is done. And that’s why I really think that the GP is just the right interface. And that’s I think the most important link in this position.
	5	CS32M: For example, is a heart failure therapy that the cardiologist has administered out of his own ambition really the optimal solution? Or is it rather necessary to keep the patient free of pain? And for individual specialists, of course, all this is hard to judge. In fact, it’s partially up to the GP or the internist to consider what’s actually best for this patient, here and now.
CSs’ role in deprescribing is well-defined and limited (CS, GP)	6	CS14M: I am totally responsible for a medication that I have prescribed, of course and on the basis of my knowledge -as far as I have some- for the other medications (…) But our policy is: repeat prescriptions, apart from a few exceptions, are made by the GP.
	7	CS14M: I never deprescribe non-cardiologic medication independently without checking with the GP. (…) Because I as a specialist in case of doubt never will have as much information as the GP who knows his patient for 20 years.
CPs should act as supporting second-line force in deprescribing (CP, GP)	8	CP21M: I once had a patient who had received a prescription for haloperidol from his psychiatrist and increasing amounts of madopar from his neurologist. The reason is obvious, right? One doctor sedated him, the other fought the side effects. That’s a true classic. When we in the pharmacy see this, we of course have the duty to bring these two together.
	9	CP12F: We have an obligation to give counsel and we must check interactions.
	10	CP11F: It actually is our profession to explain to somebody what the doctor has prescribed. We shouldn’t talk him out of it.
	11	CP21M: We don’t have the expertise. We can’t answer actual medical questions. We must not, too! We can’t. Because we haven’t studied it. We can’t make a diagnosis.
	12	GP13M: The primary responsibility for polypharmacy and prescriptions actually lies with the doctors. And I need the pharmacists as second-line force, sort of. Because when I see a medical indication and prescribe an antidepressant but didn’t get that he already has QT-prolonging medication, then I need feedback, somebody who says: Stop! Do you know what you’re doing here? Then I receive a telefax. And I appreciate that.

Legend: *GP* General practitioner, *CP* Community pharmacist, *CS* Community specialist

**Table 2:** The domain of social influences

Social influences on deprescribing activities and medication optimization		
Belief (as expressed by professional group)	Quote No.	Quote and speaker (indicating professional group, participant ID and sex e.g. GP1M = General practitioner 1, male)
Patients’ trust supports GPs’ medication authority (GP, CP, CS)	13	GP20F: If there is trust, the people first consult their GP. Or they consult him again after visiting the specialist – well, I experience this happens more often in the rural area, because you have become a person of trust (…) Sometimes they even ask: ´the cardiologist has prescribed this, am I really supposed to take it?` Then they come to us and we discuss it.
	14	GP24F: In my experience, many colleagues from other specialties add some things to the (medication) list (…) and then the patient reads the instruction leaflet and says: ‘I’ve received a prescription for this (by the specialist) but I would like you to check whether it is compatible with my other stuff.’
Pharmacists’ involvement may undermine GPs’ authority (GP, CP)	15	CP11F: Well, I feel uncomfortable here, because generally speaking, we are obliged to support the patient’s compliance. And when we have a prescription, we shouldn’t tell the patient: ´well this may not be quite appropriate`. So basically, we should support that this is what he is supposed to take, according to the doctor.
	16	GP2F: When the patients get it that the pharmacist points out like: ‘What? Your GP prescribed cortisone? This is very harmful to you- are you sure you’re supposed to take this, or do you want to reconsider?’ Then, as a GP, you’re in a defense position.
In hierarchy with specialists, GPs come off as inferior (GP)	17	GP4M: I, as humble little GP, didn’t just decide: well, cardiology is recommending this, but I say I’ll deprescribe it. I mean, somehow it’s like David versus Goliath. That’s how you feel like, somehow. And then something happens and then it’s, ‘yeah, you little prick – why have you done that?’ Right?
	18	GP9M: If you’re isolated from the world [at the country site], and have this unique feature, and it’s every Tom, Dick and Harry- That’s all very well, but then, whatever the ward physician is saying is much more relevant and important.
	19	GP1M: The specialists are always the ones who know better. And the GP must do as the specialist says. But it does not have to be like that! (…) I could imagine that general medicine becomes the queen of medicine and in the end she is the one who decides. And then it’s just tough luck for the ear, nose and throat specialist because he has a lower position in the hierarchy. (…) Currently, the opposite is true.
	20	GP3M: In the nursing homes, there’re many [psychiatrist] colleagues who look after the geriatric patients. And we totally stay out of this. I once deprescribed a medication at my own judgement (…) and this caused some major trouble. He [the psychiatrist] said, he was in charge and I should stay out of his therapy. So I stay out of it! [the other medications] -that’s my job. And he does the psychiatric medications.
Specialists exert pressure to continuing prescribing (GP)	21	GP8F: This problem occurs every time different specialty groups are concerned with one patient. Right? So, an orthopedist, a cardiologist, (…) a psychiatrist, and every physician prescribes what he would like to, or thinks to be necessary. And if you asked them right now, each one of them would say: ´Well, the Vitamin D and the calcium, he really needs both of these!`
	22	GP6F: There’s a certain attitude we (GPs) need in front of the practitioners all around [when deprescribing]. Otherwise oneself is always the penny-pincher, the one who makes plans and talks a lot, and the one who’s responsible for it in case it’s not prescribed again.
	23	GP6F: (about deprescribing a high-cost medication): No-one deprescribes it. Because nobody wants to be the one who did it (…) GP10M: But that’s what we have been discussing earlier: never change a running system!

Legend: *GP* General practitioner, *CP* Community pharmacist, *CS* Community specialist

**Table 3:** The domain of reinforcement

Reinforcement to deprescribing activities and collaboration		
Belief (as expressed by professional group)	Quote No.	Quote and speaker (indicating professional group, participant ID and sex e.g. GP1M = General practitioner 1, male)
GPs are reprimanded for deprescribing actions (GP)	24	GP13M: The biggest problem for me is the contact with other specialists, especially cardiologists. When I deprescribe a 90-years old patient’s statin (…), the guy [cardiologist] rips me into shreds, this idiot GP who doesn’t know the first thing, deprescribing the statin! He could die from this AND get a heart attack and so on. Then it’s difficult. And when I know this I won’t deprescribe anything. Because, this scolding -I mean, I can take a lot. But at some point, I need to draw a line.
CP’s medication optimization efforts are not valued (CP, GP)	25	CP19F: In 80% of the cases, one is treated with disrespect for this kind of feedback [on interaction checks]. I had it only once in my career I heard a doctor saying ‘I appreciate your call.’ (…) Once! In 20 years!
	26	GP10M: I receive a fax from the pharmacist every time something doesn’t fit together (…) this know-it-all fax: here, look, you’ve prescribed the wrong stuff together again (…) when you’ve thought it through and now there’s this fax …
	27	GP13M: When I see a medical indication and prescribe an antidepressant but didn’t get that [the patient] already has QT-prolonging medication, then I need feedback, somebody who says: Stop! Do you know what you’re doing here? Then I receive a telefax. And I appreciate that.
	28	GP17M: How do we manage all this without offending anybody? The pharmacist’s got the expertise, but the physician doesn’t want anybody to interfere.
	29	GP4M: I’m not crazy about pharmacists, because it’s such a giant commerce. I don’t believe they represent my key contact for deprescribing strategies or such things, because they want to sell it [the medications].
	30	CP12F: Every unit of medications less prescribed means for the pharmacist: Less sales. Our salary is measured by unit volume! Really! The better we consult, the more we cooperate with you, the less profit we make. In other words: we do the right thing, and we get less for it. And that’s our core problem!

Legend: *GP* General practitioner, *CP* Community pharmacist, *CS* Community specialist

**Table 4:** The domain of environmental context and resources

Environmental context and resources to deprescribing and medication optimization		
Belief (as expressed by professional group)	Quote No.	Quote and speaker (indicating professional group, participant ID and sex e.g. GP1M = General practitioner 1, male)
Reimbursement systems impede deprescribing activities (CP, CS, GP)	31	CP12F: We have an obligation to give counsel and we must check interactions. We have already received this mandate and we are penalized if we don’t comply with it. But unfortunately, it is an additional expense and really takes time, but we are not being refunded for this. That’s the great problem.
	32	CP12F: We just needed the possibility to provide consulting hours in which patients can come and ask questions about things that we have noticed. But we would also need some kind of monetary compensation for this consultation. And principally this could also reduce the doctor’s burden.
	33	CP12F: Paid work is transformed into goodwill. (…) That is the core problem. CS14M: I believe this, too. (…) The pharmacist, just like the physician, invests his time and work (in medication optimization) without getting any compensation for it. (…) Of course there is a problem at that point.
Lack of managerial resources impedes collaboration (CS, GP)	34	CS32M: In these times when we’re all very busy, (…) nobody really has time to pick up the phone and call a colleague. We really should communicate more closely and continuously with the GPs, because I think they should be in charge of defining which medication is most important for the patient whom they know from a holistic view (…) But we don’t have enough time for all this (…) And that makes it really difficult.
	35	CS33M: We not only work together with three GPs, but probably there are rather more than a hundred who we cooperate with and everyone is of a particular kind. One doctor makes a fuss if we discontinue a medication, the other one does if we don’t. One doesn’t want us to prescribe a certain medication, he wants to do it himself, the other is upset if we haven’t prescribed it. And to know all these attitudes of the respective GPs, that’s quite difficult.
Fragmentation of care impairs medication optimization (CS, GP, CP)	36	GP1M: Today a patient’s wife gave me a box full of mediations. I had tried to withdraw zolpidem from her husband (…) and there were 50, 60 medications in there, most of which I never had prescribed. That was stuff from colleagues, right? I had never received any letter about these medications! Which means, I wasn’t the only one who had prescribed zolpidem! He also had visited two psychiatrists.
	37	CS14M (on double-prescriptions): The reason is that we often don’t receive information on the medication! So I would say, 4/5 of my patients either don’t bring their medication plan or don’t know what they’re taking at all! In this case we just call the GP, ask: ´what does this patient take, anyway?` (…) The people themselves don’t know!
	38	CP21M: If someone comes in and buys an OTC medication which doesn’t match with his medication -especially if he’s taking 5–7 different meds- you would have the chance to check that. But (…) it’s difficult for us because we don’t know what else he is taking.
	39	CP11F: … if we in the pharmacy would receive a signal so we could all pull together. Because of course we have no idea what you (GPs) are thinking. And us telling the patient the opposite of what you’ve said, that’s the worst that can happen (…) If we counteract each other, we’ll get it all wrong. But if we knew: okay, you are having the same problem, that pantoprazole is a requested medication, or whatever, then- [cooperation would work]
Existing resources are not fully utilized (CP, GP, CS)	40	CP19F (on who is the patient’s central contact-point): I think, in regard to application and effect, the pharmacy is, just because there is more time for this. And it’s for free. We may always answer all questions, the pharmacy is open
	41	CP21M (on non-compliance): I think we recognize such things even better than the doctor, because the patient is too shy to tell the doctor. CP19F: I think the psychological barrier is easier to overcome in our case. At our place, one rather talks about that.
	42	GP13M: Surprisingly they [the patients] all come to you! They rarely call on us. If I say: ´Please bring all your medications`, the next time they have already forgotten to bring them. So there seems to be a higher affinity towards talking to the pharmacist than to me as a physician.
	43	GP8M (on CP-medication reviews): I really appreciate this, because often I don’t know what kind of antidepressants and stuff everybody has [prescribed].
	44	GP18M: Well what I think works well with the pharmacy: they just have a different software for medication interactions (…) GP20F: Actually I always thought this was quite helpful.
	45	CP12F: It would be awesome if there was a compatible software in the doctor’s office as well as in the pharmacy, for passing information back and forth, making it possible to read the medication plan, check it and have feedback on it.
	46	CS14M (on exchange of medication- and patient information): Actually that’s the good thing about a proper referral. And that’s why I’m actually sad that we don’t have the so-called practice fee anymore. Because in these days the patients at least came after a referral. Now there are 90% without a referral.
	47	CP21M: I really think that the GP is the best interface. And the most important link in this position. And I actually think that everyone should be obliged to visit their GP before they see a specialist!

Legend: *GP* General practitioner, *CP* Community pharmacist, *CS* Community specialist

## Results

**Fig. 1:**
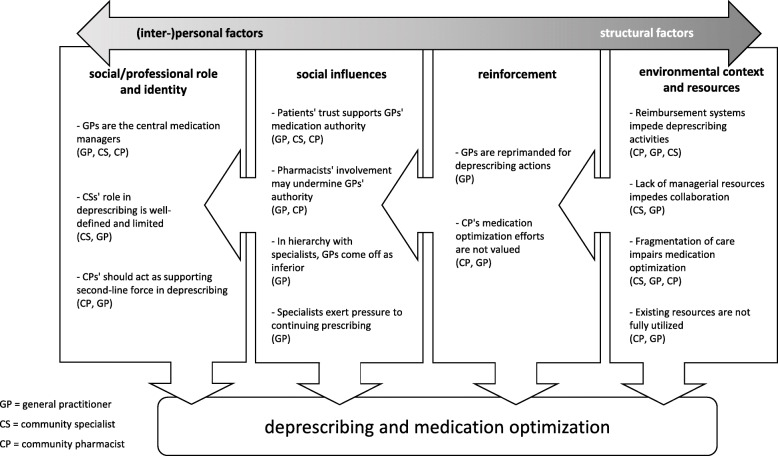
Theoretical domains to deprescribing and medication optimization. Legend: The theoretical domains social/professional role and identity, social influences, reinforcements, and environmental context and resources impact on one another as well as on deprescribing and medication optimization

The identified domains and their associated belief statements are summarized in Fig. 1. As illustrated, the four domains may be allocated on a continuum of individual and structural factors influencing joint deprescribing. Beside affecting deprescribing and medication optimization activities in a direct manner, the domains even impact on one another. In the following sections, the domains will be explored in greater detail by means of their corresponding belief statements. Despite this conceptualization, however, even conjunctions between beliefs of the different domains exist.

### Professional role and identity of stakeholders

The central themes in all discussions referred to the domain of professional roles and identity as related to deprescribing. Role understandings of each stakeholder group built on beliefs about deprescribing tasks and responsibilities, but also about limitations to such duties. Particularly, the role of GPs in deprescribing was discussed.

#### GPs are the central medication managers

The role of GPs in deprescribing was unanimously perceived as central agent by participants from all three represented professional groups (Table 1, quotes (Q) 1–5). This role was described as ‘central manager’ of medication and entailed responsibility for medication reconciliation (Q2,5). Reconciliation in this respect denoted gathering information about all medications actually being prescribed by different providers, as well as about over-the-counter (OTC) medications purchased by the patient herself, and checking them for inconsistencies or incompatibilities. Also, within a broader medication management, GPs were considered responsible for monitoring repeat prescriptions and prioritizing medications according to individual patients’ needs (Q5,6). Notably, all these tasks would entail deprescribing as a potential consequence. The supremacy in the overall medication management decisions was justified by the participating specialists with GPs’ broad knowledge about patients as well as their function as ´interface` between providers (Q3,4).

#### CSs’ role in deprescribing is well-defined and limited

In contrast to GPs’ overarching medication responsibility, participating specialists portrayed their tasks in medication management and deprescribing as rather clear-cut and confined.

Perceived duties concentrated on monitoring and discontinuation of CSs’ own prescriptions with reference to lacking patient- and medication information necessary for further deprescribing, on which CSs and GPs agreed (Q6,7).

#### CPs’ should act as supporting second-line force in deprescribing

For pharmacists’ part in medication management and joint deprescribing activities, the identified belief statement included both wide-stretching duties on the one hand, and role limitations explained by lacking authority on the other hand. Hence, while duties encompassed conducting medication reviews on medication interactions and prescription errors, as well as counselling patients on diverse medication-related matters (Q8,9), CPs clearly restrained any direct deprescribing task by appealing to their lack of mandate for this:

CP21M: *“We don’t have the expertise. We can’t answer actual medical questions. We must not, too! We can’t. Because we haven’t studied it.”* (Q11)

In this sense, CPs portrayed their role limitations in deprescribing not as themselves refusing greater involvement, but rather as external restrictions in terms of skills-based and legal demarcations to their professional terms of reference. This opinion was shared by several GPs who appreciated CPs’ medication reviews as beneficial reminders and, like one GP coined it, could envision a ‘first- and second-line task division’ in deprescribing (Q12).

### Social influences on professional roles and deprescribing collaboration

The second domain that emerged in the discussions designates diverse social influences which impact on the professionals’ role and deprescribing tasks. Here, the beliefs about patients’ trust as supporting GPs’ medication authority, CPs’ potential of undermining this authority, a hierarchy between GPs and CSs and social pressure to continue prescribing manifested.

#### Patients’ trust supports GPs’ medication authority

GPs’ predominant position in medication management and deprescribing tasks was reported to further receive support by patients’ conferral of trust (Table 2, Q13–14). The status as central person-of-trust for patients was appraised by all stakeholder groups. For long-lasting physician-patient relationships as well as in rural areas, GPs reported even being assigned superiority over CSs (Q18). This superiority would manifest in patients’ requests for guidance on medication prioritization or for reappraisal of CSs suggestions and prescriptions.

#### Pharmacists’ involvement may undermine GPs’ authority

CPs’ involvement in medication evaluation was perceived to negatively influence polypharmacy management by threatening GPs’ authority. Especially within the scope of patient counselling, both GPs and CPs themselves expressed concerns about pharmacists providing deprescribing messages or questioning prescriptions. They remarked that this could both endanger patient compliance, undermine the physician’s authority and jeopardize a trustful doctor-patient relationship (Q15,16).

#### In hierarchy with specialists, GPs come off as inferior

Remarkably, the affirmative connotations that CPs, CSs and supposedly even patients attributed to GPs’ professional role stood in harsh contrast to a belief expressed by participating GPs themselves. At numerous occasions, GPs reported a feeling of inferiority and lack of authority towards specialist providers (Q17):

*GP4M: “I, as humble little GP, didn’t just decide: well, cardiology is recommending this, but I say I’ll deprescribe it. I mean, somehow it’s like David versus Goliath. That’s how you feel like, somehow.” (Q17, continued).*

Despite some participants challenging this belief (Q19), GPs generally expressed that contact with specialist providers would exert a rather negative impact (Q17–20). The perceived hierarchy was mentioned to acuminate at the hospital setting, as GPs felt that greater importance was routinely assigned by patients to directives of ward specialists (Q18).

Importantly, dissonances in perceived authority could even manifested in intergroup conflicts. Most often, these centered around the responsibility for specialist-medication (Q20). Hence, while there was mention of CSs’ expectation about GPs to take on their repeat prescriptions (Q6), decision-making power for stopping the medication would not always be transferred. Apart from demarcating their sphere of authority, specialists’ claim for sovereignty over ´their` medications even prompted GPs to ´totally stay out of` specialists’ medication (Q20). Hence, lack of regulations and agreements between GPs and specialists on who should initiate and stop prescriptions evoked uncertainties and an inertia to deprescribe.

#### Specialists exert pressure to continuing prescribing

Specialists’ claim for sovereignty over medications was mentioned to not only impede GPs’ deprescribing efforts, but also exert social pressure to continue prescribing. As specialists would routinely prioritize their specialist-medication over the remaining (Q21), this would both spur unnecessary polypharmacy and foster a culture of re-prescribing. In light of this, GPs’ professional identity was threatened to get stigmatized of parsimonious medication management. Since deprescribing would save health-care expenditures of unnecessary and expensive medications, GPs worried to get alleged with financial motives for deprescribing and receive a bad image as ´penny pincher` in front of involved providers (Q22). In the same FGD, Q23 reflects a wish of not standing out negatively by ´changing a running system` which once again expresses social influences to continue prescribing. If GPs anyhow decided to follow an assiduous deprescribing agenda, it was deemed necessary for them to develop a firm ´attitude` towards other providers, entailing resistance of getting stigmatized (Q22).

### Reinforcements to joint deprescribing action

Among the abundance of deprescribing influences mentioned in the FGDs, two distinct beliefs about reinforcements emerged. While a belief about negative sanctions was attributed to GPs’ interaction with specialists, lacking incentives and reward were discussed for pharmacists’ efforts in medication optimization.

#### GPs are reprimanded for deprescribing actions

In addition to the above reported role conflicts about authority and hierarchy, tensions between GPs and specialists even reached a level of verbal aggression and reprimand which antagonized further deprescribing attempts:

GP13M: *“( …*) *When I deprescribe a patient’s statin ( …*), *the guy (cardiologist) rips me into shreds, ´this idiot GP who doesn’t know the first thing, deprescribing the statin!` He could die from this AND get a heart attack and so on. Then it’s difficult. And when I know this I won’t deprescribe anything. Because, this scolding -I mean, I can take a lot. But at some point, I need to draw a line.”* (Q24).

Facing suchlike conflicts, the same GP pointed out the cooperation with specialists as being the ´biggest problem` when deprescribing (Table 3, Q24). In front of perceived punishments by specialists, GPs expressed feeling discouraged from medication optimization efforts, which, by themselves, were judged time-consuming and sparsely refunded. At a general, there was no mention of positive reinforcements to deprescribing activities among GPs.

#### CPs’ medication optimization efforts are not valued

Lack of positive reinforcements even condensed in a belief about pharmacists’ efforts in medication optimization. Apart from the above-mentioned reservation about CPs’ patient counselling, CPs consistently experienced that even their performance of medication reviews would receive none, or negative feedback from GPs. One CP expressed:

*CP19F: “( …*) *I had it only once in my career I heard a doctor saying ´I appreciate your call.` ( …*) *Once! In 20 years!”* (Q25).

Although GPs’ accounts on this topic were heterogeneous with some participants expressing appreciation of medication reviews, others indeed criticized them as all-knowing and challenging GPs’ competencies (Q26,27). Hence, there was indication of some GPs’ exasperation with this service due to perceptions of offense or insult to their professional skills. As one GP put it:

*GP17M: “How do we manage all this without offending anybody? The pharmacist’s got the expertise, but the physician doesn’t want anybody to interfere.”* (Q28).

Beyond the issue of professional expertise, however, some participating GPs indicated reluctance towards collaboration with CPs even at a general level. This distancing was explained with pharmacists’ dependency on financial revenues, which triggered an overall distrust about conflict of interests (Q29). While the participating pharmacists confirmed the condition of economic dependency on a sales-per-unit reimbursement, they rated it genuinely unwanted and problematic. Being dependent on sales was perceived as both burden and scorn to CPs’ moral efforts of medication optimization. Hence, as CPs felt ´doing the right thing, and getting less for it` when engaging in deprescribing collaboration, they just expressed another negative sanction (Q30).

### Environmental context and resources

The theme of reimbursement structures already alludes the fourth identified domain environmental context and resources as a deprescribing influence. Further beliefs within this domain concerned the lack of managerial resources, fragmentation of care, but also potential assets not fully utilized yet.

#### Reimbursement systems impede deprescribing activities

For CPs, not only the sales-per-unit revenues in themselves constituted a barrier to medication optimization, but rather the lack of other reimbursement for activities such as patient counselling and performance of medication reviews. They stressed the drawback of being obliged to both give medication counselling and perform medication reviews as professional assignments while not getting compensated for it (Table 4, Q31, 32). Thus, CPs felt that the health-system environment did not provide the preconditions necessary for either medication optimization nor cooperation activities. This circumstance was even affirmed by several GPs and CSs, who in their turn appealed for better remuneration of time-consuming but hitherto uncompensated polypharmacy management such as routine medication evaluation (Q33).

#### Lack of managerial resources impedes collaboration

Adjacent to lacking reimbursement structures, CSs and GPs even judged managerial resources to be deficient. Thus, CSs raised arguments about a lack of time resources, on which GPs agreed, as well as organizational capacity for closer communication and cooperation between providers (Q34,35). As to CS33M, both scarcity of time and organizational demands for successful cooperation with GPs acuminated in urban settings:

CS33M: *“We not only work together with three GPs, but probably there are rather more than a hundred who we cooperate with and everyone is of a particular kind. One doctor makes a fuss if we discontinue a medication, the other one does if we don’t ( …*)” (Q35).

Hence, specialists explained limited deprescribing collaboration by rather pragmatic and context-related causes. Nevertheless, the resulting communication deficiencies between CSs and GPs emerged as a recurrent theme in the FGDs, and both GPs and CPs highlighted the severity of its consequences in terms of medication errors like double-prescriptions or prescribing cascades (Q8,36).

#### Fragmentation of care impairs medication optimization

The strain of dealing with prescription errors resulting from deficient knowledge exchange between providers was a theme pertinent throughout the discussions and across professional groups. However, the participants expressed a belief that these information shortages were not only product of deficient communication, but exacerbated by a system-wide fragmentation of care. Information flow between care levels, they argued, was frequently disrupted or lacking, which entailed severe impediment to polypharmacy management (Q8,36). In this sense, CSs who earlier had been alleged with skewed prioritization of their specialist-medications explained their narrow prescription focus and by lack of information about overall prescriptions:

CS14M (on double-prescriptions): *“The reason is that we often don’t receive information on the medication! So I would say, 4/5 of my patients either don’t bring their medication plan or don’t know what they’re taking at all!”* (Q37, continued).

Prudent prescribing routines, hence, were deemed impossible without synthesis of prescription information across providers. To date, however, the latter completely relies on patients’ diligence to bring along their medication plan as no further cross-level medication transparency is given in Germany. Unfortunately, the cited participant’s strategy to gather missing medication information by giving a telephone call to the respective GP just conflated with earlier-mentioned time constraints.

Yet, not only CSs requested transparency about prescription (as well as OTC-) medicines. Likewise, GPs criticized lacking information on specialists’ prescriptions (Q36) while CPs demanded more detailed and routine medication information including prescription rationale to optimize counselling (Q38). Importantly, better medication transparency was highlighted to not only benefit prescription optimization, but also help attaining synchronization of medication messages towards patients, and hence preserving colleagues’ authority. If this was achieved, participants could envision CPs and GPs to ´pull together` in persuading patients for medication optimization (Q39).

#### Existing resources are not fully utilized

In contrast to the above deficits in the structural environment, the discussions also contained accounts of resources to deprescribing collaboration not yet fully utilized. In terms of information resources, the pharmacy was depicted as a place of knowledge accumulation. As many patients would stick to their local pharmacy, not only different physicians’ prescriptions were stated to run together at the CPs’, but even knowledge about OTC-medication use and patient-related information such as medication application problems, non-adherence or side-effects (Q40–42). Hence, CPs described themselves as central contact point for patients concerning medication issues, and for patient-relevant medication matters, CPs presumed being even more knowledgeable than GPs (Q41):

CP21M: “*… we recognize such things [non-compliance] even better than the doctor, because the patient is too shy to tell the doctor.”* (Q41, continued).

This rich-in-information position was explained by the low-threshold and free-of-charge character of community pharmacy-services in Germany (Q40). In line with this, even pharmacist-led medication reviews were appraised as underutilized assets. Although several GPs felt offended by this service, there was, when speaking generally, anyhow appreciation of medication reviews checks as an otherwise ‘missing link’ of information which could bridge information gaps across providers (Q43). If not for the personal feedback in medication reviews, physicians did value CPs’ software system for interaction checks and promoted increased utilization of it (Q44). Even CPs approved this, but emphasized the urge to configurate software systems uniform or compatible to permit quick data exchange between physicians and pharmacists (Q45). Given such preconditions, CPs’ judged their involvement as capable of reducing GPs’ workload (Q32).

Finally, participants even highlighted the potential of GPs as gatekeepers to specialist services as an underutilized resource (Q46, 47). As mandatory GP consultations for specialist-referrals would optimize medication transparency and avoid multiple-prescriptions or prescribing cascades, a strengthening of this role position was requested. The fact that specifically CSs and CPs advocated this strengthening of GPs’ role positioning during the focus groups may serve as promising to future joint approaches.

## Discussion

Our focus-group study on role perceptions of GPs, pharmacists and specialists on collaborative deprescribing offered a mix of homogeneous and inter-professional discussions, allowing to explore GPs’ views uninhibited, as well as in interaction with other stakeholders. Although the literature on both barriers to deprescribing [8, 10, 43] and physician-pharmacist partnerships is growing [11, 19], this study to the best of our knowledge is the first to focus on interprofessional deprescribing collaboration as rendered in accounts of GPs, pharmacists and specialist providers. This broadened scope of knowledge about GPs’, pharmacists’, and community specialists’ interaction on different levels of care constitutes a necessary but so far neglected area of research in deprescribing.

Our study revealed influences on collaborative deprescribing on a continuum of interpersonal to structural factors. On an interpersonal level, pharmacists and specialist providers were assigned secondary roles in deprescribing, leaving a predominant position to GPs as central medication manager. At the same time, GPs’ role in deprescribing was stated to get undermined by social influences like pharmacists’ deprescribing messages towards patients, specialists’ contest of power and authority as well as a culture of re-prescribing in which preservation of status quo would prove the winning strategy. Our study also revealed reinforcing influences such as social reprimands of colleague prescribers which impeded GPs’ discontinuation attempts, and lacking appreciation of pharmacists’ effort in medication optimization. On a structural level, context-factors like adverse reimbursement systems, deficient managerial resources, but also fragmentation of care entailing lacking prescription transparency were reported to antagonize medication management. While favorable assets like information resources and GPs’ gatekeeping capacities existed, these would remain underutilized.

Our findings confirm the earlier proposed importance of a continuity of care as well as of managerial and informational assets for integrated deprescribing [4, 12]. Whereas our study confirms GPs as central stakeholders for management and continuity of care [4], need for better informational and organizational resources expanded to all involved groups.

Our analysis also highlights conflicts in the positioning and authority of involved professionals, in particular for GPs and specialist providers. Among the existing literature on specialists’ impact on deprescribing, Anderson et al. [10, 44] interpret GPs’ respect for a (specialist) colleague’s skills and autonomy as a deprescribing influence. Although our results confirm a medical hierarchy as deprescribing barrier, we found its pathway of influence to diverge. Hence, other than attributing professional autonomy to specialists’ prescribing as a positively feature, our GP-participants referred to hierarchy and authority with notions of negative or even aggressive feedback from a perceived superior. Moen et al. [45] and Wallace et al. [4] alternatingly explain specialists’ impeding influence on deprescribing by GPs’ lack of insight in the former’s prescribing rationale. Again, our results partly confirm this, as deficient prescription information was mentioned as a main driver of inappropriate polypharmacy. Yet, in contradiction to the above cited, our findings did not point to greater prescription wisdom among CSs, but rather to the problem of their lacking knowledge on overall medication intake. No matter if the deficit in prescription information primarily impedes deprescribing among GPs or provokes unnecessary prescribing among CSs, our findings agree in advocating for better communication across prescribers [4, 13, 28].

In the interaction between GPs and specialists, even previous studies have asserted a culture of prescribing which promotes ´collusions of anonymity` among concurrent providers [15, 37, 46]. While this collusion of anonymity traditionally has been explained in terms of devolving responsibility [37], participating GPs in our study rather emphasized the avoidance of stigma as motive for not ´rocking the boat` by taking on deprescribing action.

Lacking communication presented as a general barrier to cooperation and deprescribing in our study. Accordingly, other research has proposed better communication to even determine functioning collaborative care approaches in GP-pharmacist partnerships [19, 47]. Herein, not only a patient’s treatment regimen should be communicated, but even the division of professional roles and responsibilities [19, 48]. As D’Amour et al. [47] state, the development of integrated practice routines in primary care involves a redefinition of boundaries between professions. While confirming the importance of role-defining processes, our study adds in showing how a lack of such role definition evokes conflicts in perceived authority and professional tasks that impede deprescribing. Also, while pharmacists in our study referred to lacking mandate and professional restrictions as limiting their involvement in polypharmacy management, specialists rather highlighted practice-based barriers like deficient organizational or time resources. As both parties indicated general willingness to increasingly engage in deprescribing collaboration, this evidence should be considered on policy-level.

The FGDs even revealed tensions in physician-CP collaboration. A systematic review of Bardet et al. [19] explains such conflicts by the overlapping of responsibilities. Accordingly, the authors propose clear definition of professional roles and their communication as key determinants in collaboration. Our findings corroborate this suggestion. However, whereas Bardet et al. [19] claim the quality of communication to be determining rather than its quantity, our findings stress the importance of structural preconditions for such communication, like the prescription transparency across providers. For CPs’ access to more comprehensive patient information including medical records this has been asserted earlier [20]. As in the German setting, another structural determinant of GP-pharmacist tension may lie in CPs’ financial conflict of interest [27], advocating for better reimbursement of patient-oriented medication optimization services.

Lastly, our study invites to even take into account the perspectives of specialist providers on collaborative deprescribing. As specialists’ prescribing repeatedly is denoted barrier to deprescribing actions [4, 33, 34, 49] or, as in our results, gets accused of skewed prioritization, the urge for transparency of overall prescriptions should be highlighted. As long as fragmented care prevents specialists from reviewing a patient’s overall medication, prudent prescribing routines remain unattainable.

### Strengths and limitations

Our FGDs were rich in content and of noticeably cooperative character. Instead of the anticipated tensions, the representatives of different professional groups engaged in enrichening exchange of experiences which created important knowledge synergy. However, response rates for CPs and SPs remained low (20 and 10%), resulting in low numbers of included representatives of these professional groups. Hence, generalizability of the obtained findings to all stakeholders cannot be assumed. Also, as we recruited members of academic research networks, we might have included overly motivated and cooperative participants, which may present certain selection bias. Our study was conducted at two departments of general practice and entailed a majority of GPs. Especially for specialists’ appraisal of GPs’ position and authority -which contrasted GPs’ own experiences of degradation- we must consider that CSs might have felt discouraged to express views on GPs as inferior in hierarchy. Lastly, the study was conducted in Germany and participant accounts are influenced by surrounding features of this specific health-system. Nonetheless, we believe that dynamics between different groups of healthcare professionals impact deprescribing attempts in any given setting and therefore render important insights for optimizing collaborative care approaches.

## Conclusions

Given the growing complexity of polypharmacy management across care setting, it is vital to examine views of role understandings and collaboration among each stakeholder group involved. Our findings show that successful collaboration on deprescribing and medication management relies on a set of distinct preconditions that emerge from the German setting, but may as well inform other health-systems. On an interpersonal level, clear definition of roles and responsibilities must be disseminated and consented on by all involved professions to foster mutual positive valuation. On a structural level, however, such role performance and collaboration require adequate reimbursement and resources, as well as a continuity of care that secures both medication transparency and functioning gatekeeping.

## Supplementary information

**Additional file 1.**

## Data Availability

The datasets used and analyzed during the current study are available from the corresponding author on reasonable request.

## Abbreviations

CP: Community pharmacist
CS: Community specialist
GP: General practitioner
FGD: Focus group discussion
F: Female
M: Male

## References

[1] Neuner-Jehle S (2013). Less is more - how to prevent polypharmacy?. Praxis (Bern 1994).

[2] Der SJ (2018). Umgang mit Polypharmazie und die Rolle der Hausärzte: dealing with Polypharmacy and the role of family practitioners. Zeitschrift für Allgemeinmedizin.

[3] Scott IA, Hilmer SN, Reeve E, Potter K, Le Couteur D, Rigby D (2015). Reducing inappropriate polypharmacy: the process of deprescribing. JAMA Intern Med.

[4] Wallace E, Salisbury C, Guthrie B, Lewis C, Fahey T, Smith SM (2015). Managing patients with multimorbidity in primary care. BMJ..

[5] Mosshammer D, Haumann H, Morike K, Joos S (2016). Polypharmacy-an upward trend with unpredictable effects. Dtsch Arztebl Int.

[6] Haefeli WE (2011). Polypharmazie. Swiss Med Forum.

[7] Marx Y (2016). Priorisierungskriterien bei Polypharmazie: Ergebnisse einer schriftlichen Befragung von Hausärzten. Zeitschrift für Allgemeinmedizin.

[8] Reeve E, Hendrix I, Shakib S, Roberts MS, Wiese MD, To J (2013). Patient barriers to and enablers of deprescribing: a systematic review. Drugs Aging.

[9] Bokhof B, Junius-Walker U (2016). Reducing Polypharmacy from the perspectives of general practitioners and older patients: a synthesis of qualitative studies. Drugs Aging.

[10] Anderson K, Stowasser D, Freeman C, Scott I (2014). Prescriber barriers and enablers to minimising potentially inappropriate medications in adults: a systematic review and thematic synthesis. BMJ Open.

[11] Cardwell K, Smith SM (2018). Clinical pharmacists working within family practice: what is the evidence?. Fam Pract.

[12] Maskrey M, Johnson CF, Cormack J, Ryan M, Macdonald H (2018). Releasing GP capacity with pharmacy prescribing support and new ways of working: a prospective observational cohort study. Br J Gen Pract.

[13] Hazen ACM, de Bont AA, Boelman L, Zwart DLM, de Gier JJ, de Wit NJ (2018). The degree of integration of non-dispensing pharmacists in primary care practice and the impact on health outcomes: a systematic review. Res Soc Adm Pharm.

[14] Avery AJ, Bell BG (2019). Rationalising medications through deprescribing. BMJ..

[15] Sawan M, Reeve E, Turner J, Todd A, Steinman MA, Petrovic M (2020). A systems approach to identifying the challenges of implementing deprescribing in older adults across different health-care settings and countries: a narrative review. Expert Rev Clin Pharmacol.

[16] Sachverständigenrat zur Begutachtung der Entwicklung im Gesundheitswesen. Bedarfsgerechte Steuerung der Gesundheitsversorgung. https://www.svr-gesundheit.de/fileadmin/user_upload/Gutachten/2018/SVR-Gutachten_2018_WEBSEITE.pdf. Accessed 5 Sep 2019.

[17] Alosaimy S, Vaidya A, Day K, Stern G (2019). Effect of a pharmacist-driven medication management intervention among older adults in an inpatient setting. Drugs Aging.

[18] Stuhec M, Gorenc K, Zelko E (2019). Evaluation of a collaborative care approach between general practitioners and clinical pharmacists in primary care community settings in elderly patients on polypharmacy in Slovenia: a cohort retrospective study reveals positive evidence for implementation. BMC Health Serv Res.

[19] Bardet J-D, Vo T-H, Bedouch P, Allenet B (2015). Physicians and community pharmacists collaboration in primary care: a review of specific models. Res Soc Adm Pharm.

[20] Hindi AMK, Schafheutle EI, Jacobs S (2019). Community pharmacy integration within the primary care pathway for people with long-term conditions: a focus group study of patients’, pharmacists’ and GPs’ experiences and expectations. BMC Fam Pract.

[21] Michot P, Catala O, Supper I, Boulieu R, Zerbib Y, Colin C (2013). Coopération entre médecins généralistes et pharmaciens: Une revue systématique de la littérature. Sante Publique.

[22] Ammerman CA, Simpkins BA, Warman N, Downs TN (2018). Potentially inappropriate medications in older adults: Deprescribing with a clinical pharmacist. J Am Geriatr Soc.

[23] Hayhoe B, Cespedes JA, Foley K, Majeed A, Ruzangi J, Greenfield G (2019). Impact of integrating pharmacists into primary care teams on health systems indicators: a systematic review. Br J Gen Pract.

[24] NHS England (2016). General practice forward view.

[25] The Commonwealth Fund (2016). 2015 international profiles of health care systems.

[26] Püllen R, Ude C (2018). Multimedikation beim älteren Patienten: Zweiter Thementag der Veranstaltungsreihe “Ärzte und Apotheker im dialog”. Hessisches Ärzteblatt.

[27] Löffler C, Koudmani C, Böhmer F, Paschka SD, Höck J, Drewelow E (2017). Perceptions of interprofessional collaboration of general practitioners and community pharmacists - a qualitative study. BMC Health Serv Res.

[28] Mehrmann L, Ollenschläger G (2014). Problemfelder und best-practice-Ansätze in der Arzneimittelversorgung an intersektoralen Schnittstellen--Eine Literaturanalyse. Z Evid Fortbild Qual Gesundhwes.

[29] Thürmann PA (2016). Medication safety-models of Interprofessional collaboration. Dtsch Arztebl Int.

[30] Korzilius H (2008). Arzneimittelversorgung: Es hakt an der Schnittstelle. Dtsch Arztebl.

[31] Britten N, Brant S, Cairns A, Hall WW, Jones I, Salisbury C (1995). Continued prescribing of inappropriate drugs in general practice. J Clin Pharm Ther.

[32] Dybwad TB, Kjølsrød L, Eskerud J, Laerum E (1997). Why are some doctors high-prescribers of benzodiazepines and minor opiates? A qualitative study of GPs in Norway. Fam Pract.

[33] Woodward MC (2003). Deprescribing: achieving better health outcomes for older people through reducing medications. J Pharm Pract Res.

[34] Schuling J, Gebben H, Veehof LJG, Haaijer-Ruskamp FM (2012). Deprescribing medication in very elderly patients with multimorbidity: the view of Dutch GPs. A qualitative study. BMC Fam Pract.

[35] Farrell B, Tsang C, Raman-Wilms L, Irving H, Conklin J, Pottie K (2015). What are priorities for deprescribing for elderly patients? Capturing the voice of practitioners: a modified delphi process. PLoS One.

[36] Spinewine A, Swine C, Dhillon S, Franklin BD, Tulkens PM, Wilmotte L (2005). Appropriateness of use of medicines in elderly inpatients: qualitative study. BMJ..

[37] Kouladjian L, Gnjidic D, Reeve E, Chen TF, Hilmer SN (2016). Health care practitioners’ perspectives on Deprescribing anticholinergic and sedative medications in older adults. Ann Pharmacother.

[38] Michiels-Corsten M, Gerlach N, Schleef T, Junius-Walker U, Donner-Banzhoff N, Viniol A (2020). Generic instruments for drug discontinuation in primary care: a systematic review. Br J Clin Pharmacol.

[39] Hsieh H-F, Shannon SE (2005). Three approaches to qualitative content analysis. Qual Health Res.

[40] Atkins L, Francis J, Islam R, O'Connor D, Patey A, Ivers N (2017). A guide to using the theoretical domains framework of behaviour change to investigate implementation problems. Implement Sci.

[41] Islam R, Tinmouth AT, Francis JJ, Brehaut JC, Born J, Stockton C (2012). A cross-country comparison of intensive care physicians’ beliefs about their transfusion behaviour: a qualitative study using the theoretical domains framework. Implement Sci.

[42] Francis JJ, Stockton C, Eccles MP, Johnston M, Cuthbertson BH, Grimshaw JM (2009). Evidence-based selection of theories for designing behaviour change interventions: using methods based on theoretical construct domains to understand clinicians’ blood transfusion behaviour. Br J Health Psychol.

[43] Reeve E, Shakib S, Hendrix I, Roberts MS, Wiese MD (2014). Review of deprescribing processes and development of an evidence-based, patient-centred deprescribing process. Br J Clin Pharmacol.

[44] Anderson K, Foster M, Freeman C, Luetsch K, Scott I (2017). Negotiating “Unmeasurable harm and benefit”: perspectives of general practitioners and consultant pharmacists on Deprescribing in the primary care setting. Qual Health Res.

[45] Moen J, Norrgård S, Antonov K, Nilsson JLG, Ring L (2010). GPs’ perceptions of multiple-medicine use in older patients. J Eval Clin Pract.

[46] Smith SM, O’Kelly S, O’Dowd T (2010). GPs’ and pharmacists’ experiences of managing multimorbidity: a ‘Pandora’s box’. Br J Gen Pract.

[47] D'Amour D, Ferrada-Videla M, San Martin Rodriguez L, Beaulieu M-D (2005). The conceptual basis for interprofessional collaboration: Core concepts and theoretical frameworks. J Interprof Care.

[48] Dolovich L, Pottie K, Kaczorowski J, Farrell B, Austin Z, Rodriguez C (2008). Integrating family medicine and pharmacy to advance primary care therapeutics. Clin Pharmacol Ther.

[49] Gale C, Baldwin L, Staples V, Montague J, Waldram D (2012). An exploration of the experience of mental health service users when they decide they would like to change or withdraw from prescribed medications. J Psychiatr Ment Health Nurs.

