# Supplementary material for: Professional roles of general practitioners, community pharmacists and specialist providers in collaborative medication deprescribing - a qualitative study
Source: BMC Fam Pract. 2020 Sep 4;21:183. doi: 10.1186/s12875-020-01255-1 (PMC7487755; doi:10.1186/s12875-020-01255-1)
Supplement: Supplementary file 1 — Additional file 1. [file 12875_2020_1255_MOESM1_ESM.pdf]

Supplemental material

Topic guide for focus group discussions

<u>Provision of case vignette:</u>

´84-year old male patient, lusty, many clinical conditions and drugs (about 10). Patient is a typical case of less medication being desirable, and you think about deprescribing.`

Target information	Possible questions
**1. Experiences and attitudes deprescribing**	Which experiences do you have with deprescribing of medication?<br><br>What are the challenges in deprescribing?
2. **Motivation and situations for deprescribing**	What is leading you to think about deprescribing medication?
**a. Trigger situations**	What if the patient in the vignette would…<br><br>… be in residence care home?<br>… be discharged from hospital?<br>… get a palliative diagnosis?<br>... consult you for a trivial reason like cough or DMP?
**b. Patient characteristics**	Would the case (vignette) differ if the patient was demented or in need of care?<br><br>Are there other patient characteristics that influence your decision?
**c. Reasons /Motivation**	What motives/reasons do you have for deprescribing?
**d. Initiative**	Who is taking initiative on deprescribing? You, the patient or others?
**3. Heuristics** (rules of thumb)	What do you think when seeing such a long list of drugs? (on flipchart)<br>What considerations do you undertake?
**4. Substance groups**	In case vignette: which medicines would you deprescribe? Is there a difference in deprescribing allopurinol or simvastatin/ASA? Why?
a. **Risk-benefit weighting**	What do you consider and weigh? Risk/benefit?
**5. Emotions**	We would like to hear about your feelings in deprescribing decisions. Do they play a role? How do you cope with doubts and fears?<br><br>Provocative: What if you learned that the patient, after your deprescribing, had suffered an adverse event (e.g. apoplexy after ASA)?<br><br>Do you consider/fear suchlike? What would help you in these situations?

6. **Doctor-patient interaction**	If you consider it possible for medical reasons to deprescribe a drug: to what extent do you include the patient in that decision?<br>What are your experiences with that?
**a. Interaction with other stakeholders**	Who is responsible for a patient`s drugs?<br>Do you have contact with other practitioners in this matter? Specialists? Pharmacists? Care-givers?<br>What should contact look like? Who should be responsible?
**b. Risk-Benefit**	Do you discuss the pros and cons of deprescribing a medication with the patient?
**c. Conflicts**	What happens in case of diverging views between stakeholders on deprescribing? What happens if the patient expresses doubts or fear?
7. **Implementation of deprescribing attempt**	Imaging the decision of deprescribing had been made (case of e.g. citalopram or allopurinol).<br>How in detail would you proceed practically?
**a. Safety-netting**	Would you monitor the patient post-deprescribing? How?
**b. Undesired Outcome**	Do you have strategies for undesired course / outcome of deprescribing attempts?
**c. Other stakeholders**	Who is important (helpful/hindering) for implementing the deprescribing approach other than the patient? What experiences do you have in collaboration with other colleagues/ specialists in deprescribing?
**8. Wishes**	What would facilitate deprescribing approaches for you in future? What do you need? What do you miss?
